# Evaluation of accuracy and performance of self-reported HIV and antiretroviral therapy status in the Nigeria AIDS Indicator and Impact Survey (2018)

**DOI:** 10.1371/journal.pone.0273748

**Published:** 2022-08-29

**Authors:** Ibrahim Jahun, Akipu Ehoche, Moyosola Bamidele, Aminu Yakubu, Megan Bronson, Ibrahim Dalhatu, Stacie Greby, Chinedu Agbakwuru, Ibrahim Baffa, Emem Iwara, Matthias Alagi, Olugbenga Asaolu, Ahmed Mukhtar, Akudo Ikpeazu, Charles Nzelu, Jelpe Tapdiyel, Orji Bassey, Alash’le Abimiku, Hetal Patel, Bharat Parekh, Sani Aliyu, Gambo Aliyu, Manhattan Charurat, Mahesh Swaminathan

**Affiliations:** 1 Centers for Disease Control and Prevention, Division of Global HIV and TB, Center for Global Health-Nigeria, Abuja, Federal Capital Territory, Nigeria; 2 Maryland Global Initiatives, Abuja, Federal Capital Territory, Nigeria; 3 Centers for Disease Control and Prevention, Division of Global HIV and TB, Center for Global Health Atlanta, GA, United States of America; 4 Federal Ministry of Health, Abuja, Federal Capital Territory, Nigeria; 5 National Agency for the Control of AIDS, Abuja, Federal Capital Territory, Nigeria; PLOS: Public Library of Science, UNITED KINGDOM

## Abstract

**Background:**

Data on awareness of HIV status among people living with HIV (PLHIV) are critical to estimating progress toward epidemic control. To ascertain the accuracy of self-reported HIV status and antiretroviral drug (ARV) use in the Nigeria HIV/AIDS Indicator and Impact Survey (NAIIS), we compared self-reported HIV status with HIV rapid diagnostic test (RDT) results and self-reported ARV use with detectable blood ARV levels.

**Methods:**

On the basis of responses and test results, participants were categorized by HIV status and ARV use. Self-reported HIV status and ARV use performance characteristics were determined by estimating sensitivity, specificity, positive predictive value (PPV), and negative predictive value (NPV). Proportions and other analyses were weighted to account for complex survey design.

**Results:**

During NAIIS, 186,405 participants consented for interview out of which 58,646 reported knowing their HIV status. Of the 959 (weighted, 1.5%) who self-reported being HIV-positive, 849 (92.1%) tested HIV positive and 64 (7.9%) tested HIV negative via RDT and polymerase chain reaction test for discordant positive results. Of the 849 who tested HIV positive, 743 (89.8%) reported using ARV and 72 (10.2%) reported not using ARV. Of 57,687 who self-reported being HIV negative, 686 (1.2%) tested HIV positive via RDT, with ARV biomarkers detected among 195 (25.1%). ARV was detected among 94.5% of those who self-reported using ARV and among 42.0% of those who self-reported not using ARV. Overall, self-reported HIV status had sensitivity of 52.7% (95% confidence interval [CI]: 49.4%–56.0%) with specificity of 99.9% (95% CI: 99.8%–99.9%). Self-reported ARV use had sensitivity of 95.2% (95% CI: 93.6%–96.7%) and specificity of 54.5% (95% CI: 48.8%–70.7%).

**Conclusions:**

Self-reported HIV status and ARV use screening tests were found to be low-validity measures during NAIIS. Laboratory tests to confirm self-reported information may be necessary to determine accurate HIV and clinical status for HIV studies in Nigeria.

## Introduction

In 2014, to spur the global community toward ending HIV, the Joint United Nations Programme on HIV/AIDS (UNAIDS) launched the 90-90-90 targets to achieve by the year 2020: 90% of all people living with HIV (PLHIV) know their HIV status; of these, 90% are receiving sustained antiretroviral therapy; and of these, 90% have viral suppression **[1].** To monitor these targets, data on self-awareness of HIV status among PLHIV are critical; population-based HIV surveys collect these data systematically **[2].** The objective of the Population-based HIV Impact Assessments (PHIAs) is to estimate progress toward the 90-90-90 targets and program impacts per UNAIDS guidance **[3].**

Self-reported antiretroviral drug (ARV) status is now a common question in HIV population-based surveys **[4, 5]** and is a simple, efficient, and cost-effective method for assessing the prevalence of antiretroviral therapy (ART) use, though the validity of self-reported ARV status has not been well characterized **[6].** Survey respondents might not reliably provide information about their HIV or ART status or might provide information based on tests conducted in the past which might not always be accurate, and inaccurate data based on self-reported HIV status or treatment would affect the accuracy of the 90-90-90 coverage estimates, thereby misleading interventions **[7]**. To supplement self-reported ARV use, PHIA surveys have incorporated detection of ARV levels in blood specimens from all respondents who screened HIV positive. In 2018, Nigeria adapted the PHIA survey approach and conducted the Nigeria HIV/AIDS Indicator and Impact Survey (NAIIS), one of the largest population-based HIV surveys (with about 250,000 respondents) **[8]**.

Screening tests are measured against gold-standard diagnostic tests that ideally should have a score of almost 100% for all performance measurements (sensitivity, specificity, positive predictive value [PPV], and negative predictive value [NPV]). Such gold-standard tests are also expected to be the best performing tests available; however, sometimes programs must use the best available tests under reasonable conditions, known as imperfect or alloyed gold-standard tests **[9].** Self-reported HIV status (as a screening test) during NAIIS was compared with HIV biomarker screening using rapid diagnostic tests (RDT) based on the Nigeria testing algorithm (which is an imperfect gold standard: 95.0% specificity, 99.3% sensitivity, 100% PPV, and 100% NPV **[10]**); PCR tests to detect HIV DNA/RNA are the gold-standard diagnostic test for HIV **[11].** To ascertain the accuracy and performance of self-reported HIV status and ART use in NAIIS, we compared self-reported HIV status with HIV rapid diagnostic tests and self-reported ARV use with detectable blood ARV levels using liquid chromatography-tandem mass spectrometry (LC/MS/MS). The accuracy of the difference between the self-reported HIV status and the RDT might be an underestimation when compared against imperfect gold-standard RDT, known as imperfect gold-standard bias **[12].** In the case of self-reported ARV use, LC/MS/MS was used as the gold standard to test the validity of the self-reported ARV use. LC/MS/MS is an efficient and sensitive method for evaluating ARV levels in biological samples **[13].**

Our findings provide additional insights to the usefulness of including self-reported HIV and/or ARV questions in routinely conducted population-based HIV surveys for periodic monitoring of 90-90-90 coverage.

## Methods

### Ethical approvals

This evaluation was supported by an approved protocol–the Nigeria AIDS Indicator and Impact Survey (NAIIS). Informed consent was obtained from all participants, and the protocol was approved by CDC IRB, University of Maryland Baltimore IRB, and Nigeria Health Research Ethics Committee.

### NAIIS methods

NAIIS methods have been described elsewhere. The NAIIS sample design was a stratified multistage probability sample design, with strata defined by the 37 states of the country. The population of inference for NAIIS was comprised of the de facto household population. The de facto population was comprised of individuals who were present in households, i.e., slept in the household, on the night prior to the household interview **[8].** Adult participants (18 years and above) and emancipated minors (15–17 years) provided written informed consent to the interview and biomarker tests. Children aged 15–17 years were asked for assent to the interview and biomarker components after permission was granted by their parents or guardians. During the survey, participants were asked if they had ever been tested for HIV. If so, participants were asked about when and where the test was done and the result of the test. Participants were asked a series of questions about their HIV care-seeking behaviors in addition to whether they had ever received ART, the months and year when ART was initiated, and whether they are currently receiving ART. Positive responses to ever been on ART or currently on ART were both compared to the outcome of blood ARV detection.

HIV rapid tests were conducted based on the Nigerian serial testing algorithm **[14].** Participants with a non-reactive result on the HIV-screening test were reported as HIV negative. Individuals with a reactive screening test result had an RDT test repeated and confirmed with Geenius HIV 1/2 confirmatory assay. Those with a reactive result on both screening and confirmatory tests were classified as HIV positive. Participants who self-reported being HIV-positive but tested HIV-negative during the survey had their specimens further tested using DNA PCR to confirm HIV status.

Specimens were collected during survey period (July–December 2018) in the field, transported to laboratory and processed into plasma aliquots and dry blood spot (DBS) cards and stored at -80°C. ARV levels were detected in blood specimens using DBS cards by LC/MS/MS method with a limit of detection of 0.02 μg/mL for each drug and a signal-to-noise ratio of at least 5:1 for all drugs **[15].** The following drugs used in the Nigeria HIV treatment guidelines were tested: efavirenz and nevirapine (first-line regimen) and atazanavir and lopinavir (second-line regimen). These four drugs provide 100% coverage among all PLHIV receiving ARV in Nigeria. Detectable ARV biomarkers are defined as the presence of any of these four drugs in the participant’s specimen.

### Data analysis

In this analysis, only individuals who agreed to be tested for HIV were included. Performance of self-reported HIV status and self-reported ARV use as measures were assessed by comparing self-reported HIV status and the confirmed final HIV test result and self-reported ARV use and the detectable ARV in blood specimens respectively and calculating sensitivity, specificity, positive and negative predictive values as described in Table 1.

**Table 1:** Description of measures used in assessing performance of self-reported HIV status and self-reported ARV use (Nigeria HIV/AIDS and Indicator Survey 2018).

Performance measure (self-reported HIV status)	Description
Sensitivity	The proportion of participants who self-reported HIV-positive status among all those with confirmed final HIV positive test result.
Specificity	The proportion of participants who self-reported HIV-negative status among all those with confirmed final HIV negative test result.
Positive predictive value (PPV)	This is the probability that following a HIV positive self-report status, that participant will test positive for HIV after confirmatory HIV test.
Negative predictive value (NPV)	This is the probability that following a negative HIV self-report status, that participant will test negative for HIV after confirmatory HIV test.
**Performance measure (self-reported ARV use)**	
Sensitivity	The proportion of participants who self-reported ARV use among all those who have detectable ARV in their blood specimens
Specificity	The proportion of participants who self-reported not using ARV among all those without detectable ARV in their blood specimens.
Positive predictive value (PPV)	This is the probability that following a self-report ARV use, that participant will have ARV detected in their blood specimen.
Negative predictive value (NPV)	This is the probability that following a self-report of not using ARV, that participant will not have ARV detected in their blood specimen.

Abbreviations: ARV, antiretrovirals.

Data were analyzed using Stata 15 at 0.05 alpha level of significance. All results were adjusted and weighted to account for the complex survey design. The *svyset* and *anweight* command in Stata version 15 was used to set the data for sampling weights, clustering, and stratification. The *anweight* corrects for differential selection probabilities within each sample location as specified by sample design, for nonresponse, for noncoverage, and for sampling error related to the four post-stratification variables and takes into account differences in population size across study location.

UNAIDS 90-90-90 targets were estimated based on self-reported HIV-positive status and verified by ARV biomarker data. Using only self-reported status, we categorized participants as aware of their HIV-positive status if they self-reported knowing they were HIV positive before NAIIS HIV testing and as receiving ART if they self-reported ARV use. Using both self-reported and biomarker data, we adjusted self-reported HIV positive awareness and ARV use to include participants with ARV biomarkers detected in their blood specimen, even if these participants did not self-report HIV positive status or ARV use. In both sets of results, individuals who had viral load suppression but were not aware of their HIV-positive status or were not receiving ART, either by self-report or ARV biomarker data, were excluded from the numerator for the third 90 **[8].**

## Results

**Fig 1:**
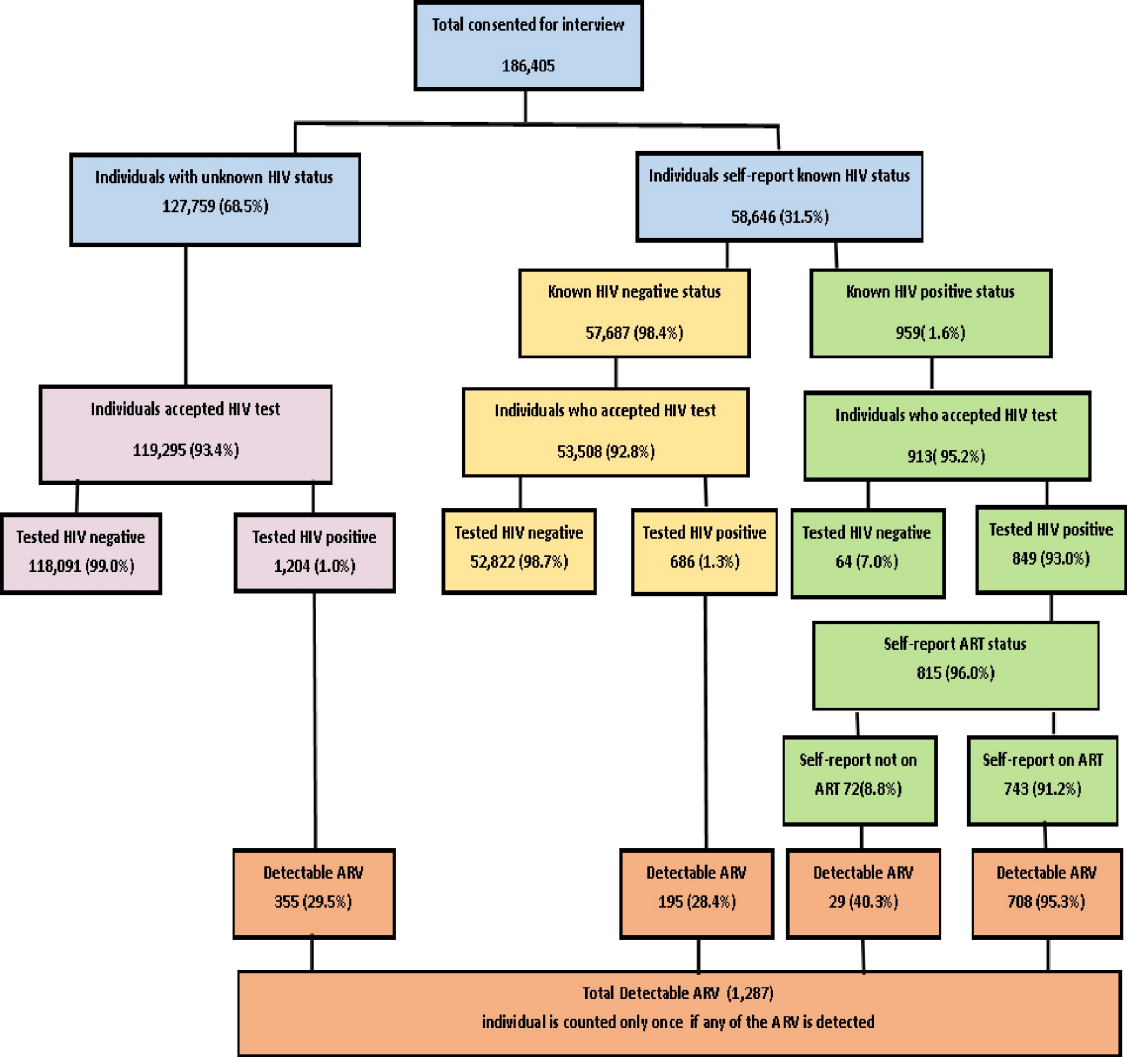
Cascade of self-reported HIV status and self-reported antiretroviral drug (ARV) use among individuals who consented for interview (percentages within the figure are unweighted). (Nigeria HIV/AIDS and Indicator Survey 2018).

Characteristics of HIV-positive study population and self-reported HIV status and self-reported antiretroviral drug (ARV) use among individuals who consented for interview during the study can be found in Table 2 and Fig 1 respectively. The proportions presented in this section are weighted proportions and therefore may not agree with values presented in Fig 1 (unweighted values). Of the total 186,405 NAIIS participants aged 15–64 years who consented for interview, 58,646 participants self-reported their HIV status of which 959(weighted, 1.5%) reported being HIV positive, and 57,687 (98.5%) reported being HIV negative. Of those who self-reported being HIV positive, 913 (94.3%) accepted an HIV test. Of these, 849 (92.1%) tested HIV positive, and 64 (7.9%) tested HIV negative via HIV RDT. All 64 persons who tested HIV negative via RDT were confirmed by DNA PCR, including the 10 who reported receiving ART. Of the individuals who self-reported being HIV negative (57,687), 686 (1.2%) tested HIV-positive via RDT, and ARV biomarkers were detected among 195 (25.1%). Overall, the HIV-positivity rate among participants who self-reported known HIV positive or negative status increased from 1.6% to 2.6% after HIV self-report status was confirmed with RDT. Of those who tested HIV-positive, 96.0% self-reported their ARV use status:743 (89.8%) reported using ARV, and 72 (10.2%) reported not using ARV. ARV drugs were detected among 94.5% of those who self-reported using ARV and among 42.0% of those who self-reported not using ARV. However, ARV were not detected among 5.5% of individuals who self-reported using ARV.

**Table 2:** Sociodemographic characteristics of individuals who self-reported HIV status and antiretroviral drug (ARV) and who tested HIV positive during the Nigeria HIV/AIDS and Indicator Survey (NAIIS 2018).

Characteristic	PLHIV in NAIIS n (weighted %)	Self-report HIV status n (weighted %)	Self-report ARV use n (weighted %)
**Total**	2,739 (100)	1,535 (100.0)	815 (100.0)
**Sex**			
Male	845 (35.9)	459 (33.9)	251 (34.0)
Female	1,894 (64.1)	1,076 (66.1)	564 (66.0)
**Age, years**			
15–19	82 (3.3)	22 (1.5)	9 (1.1)
20–24	234 (9.5)	98 (7.1)	38 (5.2)
25–29	354 (12.3)	203 (12.9)	67 (7.9)
30–34	394 (14.1)	227 (15.5)	100 (11.8)
35–39	472 (16.9)	299 (18.8)	163 (18.9)
40–44	383 (13.5)	205 (12.7)	134 (16.8)
45–49	289 (11.8)	183 (13.4)	110 (15.6)
50–54	263 (9.0)	155 (9.5)	103 (12.6)
55–59	139 (6.2)	77 (5.8)	45 (6.4)
60–64	129 (3.4)	66 (2.8)	46 (3.7)
**Age category**			
15–24	316 (12.8)	120 (8.6)	47 (6.3)
15–49	2,208 (81.4)	1,237 (81.9)	621 (77.4)
15–64	2,739 (100.0)	1,535 (100.0)	815 (100.0)
**Location**			
Urban	1,078 (44.1)	687 (48.9)	369 (49.7)
Rural	1661 (55.9)	848 (51.1)	446 (50.3)
**Education**			
No education	454 (14.9)	172 (9.5)	109 (10.6)
Primary	720 (25.0)	366 (22.2)	203 (23.4)
Secondary	1,116 (43.0)	673 (46.4)	324 (43.6
Tertiary	381 (14.1)	297 (19.7)	161 (19.6)
Other	63 (3.0)	25 (2.2)	17 (2.8)
**Wealth Qualities**			
First (poor)	317 (10.1)	117 (6.3)	64 (6.9)
Second	466 (15.5)	242 (14.3)	156 (16.9)
Third	714 (24.9)	365 (23.1)	210 (25.6)
Fourth	704 (25.8)	433 (27.9)	221 (28.1)
Fifth (wealthiest)	538 (23.7)	378 (28.4)	164 (22.5)

Abbreviations: PLHIV, people living with HIV.

In NAIIS, self-reported HIV status had a low sensitivity (52.7% [95% confidence interval (CI): 49.4%–56.0%]) with high specificity (99.9% [95% CI: 99.8%–99.9%) in determining HIV status. Individuals were more likely to report their HIV-negative status accurately than to report their HIV-positive status as indicated by NPV and PPV of 98.7% (95% CI: 98.6%–98.8%) and 92.1% (95% CI: 89.4%–94.2%), respectively (Table 3).

**Table 3:** Self-reported HIV status performance criteria (Nigeria HIV/AIDS and Indicator Survey 2018).

Characteristic	Sensitivity	Specificity	PPV	NVP
	% (95% CI)	% (95% CI)	% (95% CI)	% (95% CI)
**Total**	52.7 (49.4–56.0)	99.9 (99.8–99.9)	92.1 (89.4–94.2)	98.7 (98.6–98.8)
**Sex**				
Male	52.1 (46.4–57.6)	99.9 (99.9–100)	93.9 (89.9–96.4)	99.1 (99.0–99.2)
Female	53.0 (49.3–56.7)	99.8 (99.8–99.9)	91.3 (87.5–94.0)	98.4 (98.3–98.6)
**Age, years**				
15–19	38.9 (18.4–64.3)	99.8 (99.0–99.9)	52.1 (17.4–85.0)	99.6 (99.2–99.7)
20–24	39.2 (28.7–50.7)	99.8 (99.7–99.9)	85.6 (72.1–93.2)	99.2 (99.0–99.4)
25–29	33.6 (26.4–41.8)	99.8 (99.7–99.9)	80.0 (69.2–87.7)	98.7 (98.5–98.9)
30–34	41.1 (33.4–49.3)	99.9 (99.8–99.9)	90.4 (82.9–94.8)	98.6 (98.2–98.9)
35–39	53.5 (46.8–60.2)	99.9 (99.8–100.0)	94.4 (88.8–97.3)	98.4 (98.1–98.7)
40–44	70.2 (62.4–76.9)	99.8 (99.5–99.9)	92.4 (81.1–97.2)	99.1 (98.8–99.3)
45–49	60.3 (51.5–68.4)	99.9 (99.7–100.0)	96.4 (89.3–98.9)	98.2 (97.6–98.6)
50–54	68.0 (59.0–75.8)	100.0 (100.0–100.0)	96.8 (80.4–99.6)	98.3 (97.5–98.8)
55–59	56.0 (42.9–68.4)	99.9 (99.8–100.0)	100.0 (100.0–100.0)	98.9 (98.2–99.4)
60–64	65.5 (50.8–77.8)	100.0 (100.0–100.0)	100.0 (90.6–100.0)	98.9 (98.3–99.3)
**Age category**				
15–24	39.1 (29.6–49.5)	99.9 (99.8–100.0)	77.1 (57.8–89.2)	99.3 (99.1–99.5)
15–49	50.2 (46.5–53.9)	99.9 (99.8–99.9)	90.4 (86.9–92.9)	98.7 (98.6–98.9)
15–64	52.7 (49.4–56.0)	99.9 (99.8–99.9)	92.1 (89.4–94.2)	98.8 (98.6–98.9)
**Location**	** **			
Urban	53.7 (48.7–58.8)	99.9 (99.8–99.9)	91.3 (86.5–94.6)	98.9 (98.8–99.1)
Rural	51.7 (47.4–56.0)	99.9 (99.8–99.9)	92.9 (89.7–95.2)	98.5 (98.3–98.6)
Education				
No education	62.8 (52.9–71.6)	99.8 (99.1–99.9)	91.6 (72.0–97.8)	98.6 (98.0–99.0)
Primary	54.3 (48.0–60.4)	99.8 (99.5–99.9)	89.4 (81.5–94.2)	98.2 (97.9–98.4)
Secondary	49.5 (44.6–54.5)	99.9 (99.8–99.9)	93.2 (89.8–95.4)	98.6 (98.4–98.7)
Tertiary	52.6 (45.6–59.6)	99.9 (99.9–100.0)	93.0 (87.5–96.2)	99.2 (99.0–99.3)
Other	63.5 (33.4–85.8)	99.9 (99.6–100.0)	95.2 (70.9–99.4)	99.3 (98.1–99.7)
**Wealth Qualities**				
First (poor)	59.4 (48.1–69.8)	99.7 (99.5–99.9)	83.3 (70.4–91.3)	99.0 (98.5–99.3)
Second	62.1 (53.8–69.9)	99.9 (99.8–100.0)	95.1 (90.2–97.6)	98.7 (98.3–99.0)
Third	57.1 (50.5–63.5)	99.8 (99.5–99.9)	88.6 (80.1–93.8)	98.5 (98.2–98.8)
Fourth	53.6 (47.6–59.6)	99.9 (99.9–100.0)	96.1 (92.7–98.0)	98.7 (98.5–98.9)
Fifth (wealthiest)	41.9 (35.4–48.8)	99.9 (99.9–100.0)	92.2 (85.98–95.8)	98.9 (98.6–99.1)

Abbreviations: CI, confidence interval; PPV, positive predictive value; NPV, negative predictive value

Sensitivity and PPV of self-reported HIV status improved slightly with increasing age: adolescents and young adults aged 15–29 years had the lowest sensitivity and PPV, and adults aged 40–64 years had the highest sensitivity and PPV. Sensitivity of self-reported HIV status was highest among participants in the second wealth quintiles, and lowest among those in the fifth (wealthiest) quintile. Sensitivity of self-reported HIV status was higher among participants in urban areas than those in rural areas (Table 3).

Self-reported ARV use had high sensitivity (95.2% [95% CI: 93.6%–96.7%]) and low specificity (54.5% [95% CI: 48.8%–70.7%]) in determining ARV use (Table 4). Individuals were more likely to report using ARV accurately than to report not using ARV as indicated by high PPV (94.5% [95% CI: 91.6%–99.9]) and low NPV (58.0 [95% CI: 48.8%–70.7%]). The sensitivity of self-reported ARV use improved slightly with increasing age and was highest among participants aged 40–64 years and lowest among adolescent and young adults aged 15–24 years and among adults aged 30–34 years. Sensitivity of self-reported ARV use was lowest among participants in the first (poor) wealth quintile and was highest among individuals in the wealthier fourth and fifth quintiles (Table 4).

**Table 4:** Self-reported antiretroviral drug (ARV) use performance criteria (Nigeria HIV/AIDS and Indicator Survey 2018).

Characteristic	Sensitivity	Specificity	PPV	NVP
	% (95% CI)	% (95% CI)	% (95% CI)	% (95% CI)
**Total**	95.2 (93.6–96.7)	54.5 (48.8–70.7)	94.5 (91.6–99.9)	58.0 (48.8–70.7)
**Sex**				
Male	96.9 (93.5–98.6)	35.4 (16.8–59.7)	93.4 (87.1–96.7)	55.1 (29.3–78.4)
Female	94.2 (90.4–96.6)	62.7 (48.2–75.3)	95.1 (92.2–96.9)	58.7 (42.3–73.4)
**Age, years**				
15–19	85.7 (39.0–98.3)	55.7 (7.3–95.3)	87.6 (42.8–98.5)	51.8 (6.3–94.5)
20–24	85.2 (63.2–95.1)	87.1 (44.6–98.3)	96.3 (77.4–99.5)	60.2 (27.3–85.9)
25–29	94.1 (83.4–98.0)	51.2 (22.3–79.4)	89.8 (77.3–95.8)	65.3 (29.9–89.3)
30–34	83.7 (66.6–93.0)	51.7 (23.9–78.4)	92.4 (82.8–96.9)	31.0 (11.6–60.6)
35–39	96.2 (91.0–98.5)	77.9 (53.9–91.4)	97.2 (93.1–98.9)	72.3 (46.2–88.8)
40–44	99.4 (96.1–99.9)	53.0 (25.7–83.6)	95.3 (88.9–98.1)	92.0 (56.1–99.1)
45–49	96.8 (91.7–98.8)	40.7 (9.6–81.7)	96.2 (87.6–98.9)	45.2 (13.0–82.0)
50–54	98.2 (88.1–99.7)	8.4 (0.9–48.7)	91.8 (75.5–97.6)	30.5 (2.7–87.5)
55–59	100.0 (100.0–100.0)	0	92.9 (79.0–97.8)	0
60–64	97.2 (88.7–99.4)	76.7 (20.1–97.7)	97.4 (83.4–99.6)	75.8 (25.5–96.6)
**Age category**				
15–24	85.3 (66.5–94.4)	58.9 (29.2–83.3)	94.6 (80.2–98.7)	58.9 (29.2–83.3)
15–49	94.2 (90.7–96.4)	62.0 (48.2–74.1)	95.0 (92.4–96.7)	58.2 (42.9–72.0)
15–64	95.2 (93.6–96.7)	54.5 (48.8–70.7)	94.5 (93.6–96.7)	56.0 (48.8–70.7)
**Location**				
Urban	94.6 (94.1–98.2)	58.3 (38.0–70.1)	95.7 (94.1–98.2)	52.2 (38.0–70.1)
Rural	95.8 (92.4–97.7)	51.8 (37.1–66.1)	93.3 (89.7–95.6)	63.8 (45.0–79.2)
**Education**				
No education	93.9 (78.6–98.5)	39.5 (13.5–73.3)	91.6 (81.4–96.5)	48.0 (13.2–84.9)
Primary	94.9 (88.4–97.9)	43.9 (20.6–70.1)	95.4 (90.6–97.8)	41.4 (17.6–70.1)
Secondary	95.2 (91.6–97.3)	53.7 (35.3–71.1)	92.6 (87.1–95.9)	64.5 (45.9–79.6)
Tertiary	99.4 (95.9–99.9)	80.5 (49.5–94.6)	98.2 (92.9–99.6)	92.7 (61.5–99.0)
Other	73.8 (30.7–94.7)	0	100.0 (100.0–100.0)	0
**Wealth quartiles**				
First (poor)	90.9 (79.0–96.4)	43.2 (7.9–87.1)	96.8 (87.1–99.2)	20.4 (3.7–63.4)
Second	95.9 (89.9–98.4)	24.3 (8.3–53.3)	91.8 (84.0–95.9)	40.2 (14.3–73.1)
Third	91.2 (81.9–95.9)	68.3 (44.6–85.2)	95.6 (90.8–98.0)	50.3 (27.0–73.5)
Fourth	97.8 (94.7–99.1)	44.1 (22.9–67.9)	92.4 (84.6–96.4)	74.4 (48.5–89.9)
Fifth (wealthiest)	97.3 (92.1–99.1)	77.0 (49.4–92.0)	97.4 (92.9–99.1)	76.5 (46.4–92.5)

Abbreviations: CI, confidence interval; PPV, positive predictive value; NPV, negative predictive value.

Estimated progress toward the UNAIDS 90-90-90 targets of weighted population based on self-reported HIV and ARV status were 30-90-83 versus 47-96-81 after adjusting estimates with ARV biomarker detection of all confirmed HIV-positive cases.

## Discussion

Self-reported HIV status and ARV use were found to be low-validity measures during NAIIS. A high proportion of participants who reported being tested for HIV and reported the results were confirmed by biomarker RDT test, with 7.0% false-positive and 1.3% false-negative results. The high false-positive rate is not surprising due to that self-reported HIV status was compared with the “imperfect” gold standard RDT (95.0% specificity, 99.3% sensitivity, 100% PPV and 100% NPV) having a relatively low specificity. All the 64 (7.0%) individuals who self-reported as HIV-positive tested HIV-negative using RDT were further confirmed to be HIV negative on HIV DNA PCR. Results from 11 PHIA surveys in Sub-Saharan Africa have reported similar variance and it was extrapolated that about 73,000 individuals might have been misdiagnosed as PLHIV, with about 39.7% of them likely to have been erroneously initiated on ART in the 11 countries **[16]**. Ethically, misdiagnosis of an individual as HIV-positive may have serious moral and psychological consequences which may lead to medico-legal complications **[17]** while, in monitoring progress toward UNAIDS 90-90-90 targets, it may lead to overestimation of 1^st^ 90 (and 2^nd^ 90 if initiated on ARV). HIV-negative participants were more likely to report their HIV status correctly than HIV-positive participants as confirmed by a high specificity of 99.9% and low sensitivity of 52.7% of self-reported HIV status.

Following ARV testing of blood samples from all participants who tested HIV positive, a high proportion (28.4%) of individuals who self-reported being HIV negative had detectable ARV biomarkers, indicating possible intentional concealment of their HIV-positive status. This is not surprising due to pervasive HIV stigma and discrimination in Nigeria **[18–20]**. Additionally, studies have shown HIV-positive individuals may decide to conceal their status, especially in healthcare-related activities such as surveys, due to concerns about breach of confidentiality, fear of denial of quality healthcare, and judgement from healthcare providers **[21, 22].** False-negative results lead to the underestimation of progress toward the first UNAIDS 90 target and HIV prevalence **[23]**; for example, in this study, unweighted prevalence among participants who self-reported their HIV status was 1.6% and was adjusted to 2.6% with biomarker RDT results. Factors such as stigma or seroconversion after their last HIV-negative test could explain the low sensitivity of 52.7% in our study. A similar study in rural Malawi also reported low sensitivity (38.7%) for self-reported HIV status **[24].**

Studies have shown that adolescents and children have low disclosure rates **[25]**, perhaps because they were not informed about their status by caregivers **[26]** or because they fear stigma and isolation by their peers **[27, 28]**; this may explain the low sensitivity of the screening question among adolescents and young adults in our analysis. Additionally, younger people may also have more likelihood of infections since their last test, since infection rates are higher in these age groups, leading to lower sensitivity in this age group.

In contrast to self-reported HIV status, HIV-positive participants receiving ART were more likely to self-report ARV use, probably because ARV use is considered good practice and is encouraged. This is supported by the high PPV; additionally, among participants who reported receiving ART only 5.5% had no detectable ARV biomarkers in their blood in our study, which may be due to recent poor adherence, as reported in other studies **[4]**. With 54.5% specificity, self-reported ARV use may not correctly identify individuals who are not using ARV because up to 45.5% may be misclassified as not receiving ART. It is difficult to ascertain why PLHIV may decide to conceal ARV use in survey settings after disclosing HIV-positive status. Some studies reported similar cases of disclosure of HIV status but non-disclosure of ARV use **[6, 29–31]** and suggested fear of exclusion from the survey or fear of breach of confidentiality if they had not disclosed their HIV-positive status to their partners **[32]** as possible explanations. We found that age influenced self-reported ARV use and, as with self-reported HIV status, the sensitivity of self-reported ARV use was lower among adolescents and young adults than adults. Therefore, self-reported HIV status or ARV use may need to be confirmed by RDT or ARV biomarker testing (LC/MS/MS) even though detecting blood ARV levels is expensive and requires expertise not readily available in most sub-Saharan African countries **[33–35]** including Nigeria. Inaccurate self-reported ARV use data could lead to underestimation of progress toward the second UNAIDS 90 target, as found in several studies and other PHIAs **[22, 36–38]** including NAIIS **[8]**. Underreporting progress toward the second 90 target also could affect the first and third 90 coverage estimates.

Previous studies have shown ARV biomarker data can be used to adjust self-reported HIV status by recategorizing respondents with detectable ARV blood levels as having a previous HIV diagnosis and receiving ART **[4, 36].** The inclusion of biomarkers added to reliability of 90-90-90 estimates, which support planning and implementation of effective strategies for HIV epidemic control. In our study, the 17% gap in the first UNAIDS 90 target coverage between self-reported ARV status (30%) and adjusted ARV (47%) shows that self-reported HIV status may impact the accuracy of the underlying data used to estimate progress toward the first 90 target and also reiterated the importance of ARV biomarker results in adjusting not only progress toward the second 90 target but also the entire 90-90-90 cascade. In our study, ARV biomarker results were more likely to change estimated progress toward the first 90 target than the second or third 90 targets because individuals who have already disclosed their HIV-positive status are more likely to report receiving ART. In Kenya, a similar study showed that the progress toward the first 90 target changed from 46.9% based on self-reported HIV status to 52.7% after adjustment using ARV biomarker results, to 57.5% when adjusted by undetected viral load data, and to 59.8% when adjusted by either ARV biomarker or undetected viral load data **[39].** Unlike the Kenya study, in NAIIS, we did not adjust progress toward the first 90 target by undetected viral load data because few of the respondents had undetectable viral load. Despite the complexity, cost, and lack of ready availability, ARV biomarker tests’ impact on adjustment of progress toward the first 90 target outweigh these constraints. However, use of undetectable viral load data may serve as an alternative in populations with high undetectable viral load rates because viral load testing is a major component of comprehensive ART programs and is readily available in most resource-limited settings.

Our findings are subject to at least four limitations. First, since only participants who agreed to be tested for HIV were analyzed, there is likelihood of some participants among those who didn’t accept testing to be aware of their HIV status and this might have affected the overall findings discussed. Similarly, ARV drug testing was not performed for participants with unknown or negative self-reported HIV status who had a single negative rapid test. While it may not have been feasible to test these persons for ARV drugs because of the large size of this group, some of these persons could have been on ART and it is well known that ART can down-regulate antibody production and can cause false negative HIV rapid tests [32, 40]. Second, we were unable to ascertain the accuracy of self-reported ARV use by 10 participants who had a previous misdiagnosis because ARV assays were done only if there was a confirmed HIV-positive test result. Third, participants were more likely to report accurately conditions that are regarded as good practice and were more likely to conceal conditions that may be regarded as bad practice. This limitation might have biased self-reported responses in our study. Fourth, ARV testing included only four drugs. While the four drugs tested cover the first-line and second-line drug regimens in Nigeria, some participants who self-reported taking ARV drugs may have taken ARV drugs other than EFV, NVP, AZT, and LPV. Similarly, recreational EFV use cannot be distinguished from an EFV-based ART regimen in this study. Crushing and smoking of an EFV- cannabis mixture, commonly known as “Nyaope” or “Woonga”, in certain countries like South Africa, has seen the recreational use and abuse of EFV escalate to alarming proportions [41, 42]. Self-reported HIV status and ARV use were found to be low-validity measures of HIV status and ARV use in our study and may not be useful as the only screening tests in other HIV studies in Nigeria. Biomarker tests such as RDT and LC/MS/MS tests can be incorporated in future studies to validate HIV status and ARV use. Evaluating the Nigeria HIV testing algorithm and protocols and routinely assessing the proficiency of laboratory scientists who conduct HIV RDTs could help decrease false-positive HIV results in Nigeria. A follow-up study to estimate the population of HIV-false-positive individuals on ART in Nigeria is recommended and findings from the study could serve as additional justifications for the implementation of other recommendations proposed in this study.

## Supporting information

S1 Data (CSV)
